# Study protocol: transforming outcomes for patients through medical home evaluation and redesign: a cluster randomized controlled trial to test high value elements for patient-centered medical homes versus quality improvement

**DOI:** 10.1186/s13012-015-0204-6

**Published:** 2015-01-22

**Authors:** David A Dorr, Kenneth John McConnell, Marsha Pierre-Jacques Williams, Kimberley A Gray, Jesse Wagner, Lyle J Fagnan, Elizabeth Malcolm

**Affiliations:** Oregon Health and Science University, SW Sam Jackson Park Rd, Portland, OR USA; Stanford University, Serra Mall, Stanford, CA USA

## Abstract

**Background:**

Health care in the United States is in the midst of a near perfect storm: strong cost pressures, dramatic redesign efforts like patient-centered medical homes and accountable care organizations, and a broad series of payment and eligibility reforms. To date, alternative models of care intended to reduce costs and improve outcomes have shown mixed effects in the U.S., in part due to the difficulty of performing rigorous evaluation studies that control for the broader transformation while avoiding other biases, such as organizational or clinic effect on individual patient outcomes. Our objective is to test whether clinics assigned to achieve high value elements (HVEs) of practice redesign are more likely than controls to achieve improvements in patient health and satisfaction with care and reduction in costs.

**Methods/Design:**

To prepare, we interview stakeholders, align with health reform, and propose a pilot. Participants are primary care clinics engaged in reform. Study protocol requires that both arms receive monthly practice facilitation, IT-based milestone reporting, and small financial incentives based on self-determined quality improvement (QI) goals; intervention receives additional prompting to choose HVEs. Design is a cluster randomized controlled trial over 1 year with pre- and post-washout periods. Outcomes are unplanned utilization and costs, patient experience of care, quality, and team performance. Analysis is a multivariate difference-in-difference with adjustments for patient risk, intraclinic correlation, and other confounders.

**Discussion:**

The TOPMED study is a cluster randomized controlled trial focused on learning how primary care practices can transform within health reform guidelines to achieve outcomes related to the Triple Aim.

**Trial registration:**

ClinicalTrials.gov registration: NCT02106221.

**Electronic supplementary material:**

The online version of this article (doi:10.1186/s13012-015-0204-6) contains supplementary material, which is available to authorized users.

## Introduction

United States health care costs per capita are more than 50% larger than other developed countries, yet our health outcomes lag in many respects [1]. To combat this disparity, the U.S. is engaged in a number of health reform efforts. For instance, billions are being invested in accountable care organizations (ACOs)—intended to virtually integrate health systems, align incentives, and optimize care for populations—and patient-centered medical homes (PCMHs)—intended to change the incentives and impact of primary care settings to better engage patients, improve quality, and improve care coordination [2]. However, the evidence about how to reform care in a complex and fragmented system is unclear [3,4] and the outcomes of previous efforts are mixed [5–8]. Although trial design has grown increasingly sophisticated over the last 20 years, designing and analyzing trials in the midst of large health reform efforts is still challenging. Analyses have shown that intervention definitions, contemporaneous trends, sample size estimates, and unit of analysis all confuse results [4]. In this paper, we describe the protocol of a complex cluster randomized controlled trial named Transforming Outcomes for Patients through Medical Home Evaluation and reDesign or TOPMED.

TOPMED is intended to test whether primary care clinics in Oregon engaged in both PCMH- and ACO-like redesign efforts can improve outcomes. In Oregon, coordinated care organizations are ACO-like models that bundle care for Medicaid patients, creating or adapting regional entities whose goals are to reduce cost of care while meeting benchmarks in quality and patient experience of care. Their primary approach involves care coordination, increased population management systems with stratification, and combining previously disparate systems like mental health, housing, and health systems in novel ways [9]. Similarly, for PCMH, Oregon chose a state recognition process (called the patient-centered primary care home) and payment reform through efforts like the Comprehensive Primary Care (CPC) initiative, funded through the Centers for Medicare and Medicaid Services (CMS), and primary care clinics are receiving a combination of proactive care management payments and shared savings to increase population management, care coordination, quality improvement, and engaging patients and families.

Previous evidence of the effectiveness of these models is mixed. Studies at Group Health, Geisinger, and Intermountain Healthcare demonstrated success [5,6,10], but several others, including the national demonstration project (NDP), a cluster randomized trial of 36 primary care clinics, improved quality metrics but failed to affect the key outcomes of cost, utilization, and patient experience [7]. Since the definitions of PCMHs are complex, we and others have identified key concepts—what we call ‘high-value elements’—that may help lead to success. In the Care Management Plus (CMP) trial at Intermountain, which studied the use of care managers embedded in seven primary care clinics compared to a set of matched control clinics, success factors included an integrated care manager, an IT-based population management system, training in complex illness care, motivational interviewing, self-management and goal setting, and how to address issues for older adults and patients identified at higher risk. In all, 1,440 patients with complex illness needs were referred during the 2-year study period. For similar patients with multiple chronic illnesses, hospitalizations were reduced by 24%–40%, patient satisfaction was higher, and patient health status was improved for those referred to care management compared to controls. Similarly, Brown et al. identified seven characteristics that defined the effective Medicare chronic care demonstration sites: 1) care coordinators with face-to-face contact with patients; 2) strong relationships with physicians; 3) patient education; 4) managing care transitions; 5) acting as a communications hub; 6) medication management; and 7) addressing psychosocial issues [3].

A number of these concepts—care coordination, care management, population management, use of IT, focusing on complex patients—are used in PCMH and ACO-like models, but most of the elements do not directly relate to known drivers of cost reduction [11]. Thus, in pursuing health reform, clinics have many different paths to choose to achieve transformation and potentially impact these outcomes. Assistance in transformation such as learning collaboratives and practice facilitation may help but does not appear to be sufficient to consistently achieve desired outcomes [12]. We wanted to understand if we could use practice facilitation coupled with IT and incentives to drive clinics towards more effective components. However, trial design in health reform is notoriously complex, with previous reviews demonstrating multiple biases and confounders that threaten study validity and success [12]. In addition, pragmatic trial design requires a protocol that can work in the real world; that is, where clinical teams need flexibility, to maintain autonomy and decision-making as much as possible, and are not burdened with additional study requests.

In our trial design process for TOPMED, we attempted to understand how one could overcome the weaknesses and mixed outcomes of previous trials. Building upon recent efforts, TOPMED integrates lessons from model implementation and trial design into health reform efforts while revising study design to account for biases. Thus, we create a trial aligned with these efforts to understand if a more targeted approach to primary care transformation is more effective than the general approach. We use a cluster randomized control trial design because our intervention involves quality improvement efforts at the organizational level of practice sites.

### Objectives

The objective of this study is to understand whether focused versus general quality improvement efforts in a pragmatic cluster randomized trial can facilitate improvement in patients’ health and satisfaction with care while reducing costs. The trial outcomes and hypotheses are both at the cluster and patient level.

The study has two major research questions:

1. Would a study aligned with major reform efforts with focused practice facilitation, targeted incentives, and IT-based reporting enhance quality improvement capability and likely achievement of selected health reform goals within the clinics?
2. Would a focus on a set of predetermined ‘high-value elements’ from the many choices in health reform can improve outcomes more than general quality improvement?

## Methods/Design

The proposed trial design is a cluster randomized controlled trial with a high-value element (intervention) and a general quality improvement arm (control), where randomization is at the clinic level and matching is used to minimize baseline clinic differences between arms.

### Preparation

We took several complementary approaches to explore different trial design options in the complex health reform environment for the TOPMED trial. First, we matched the trial design to the PRECIS pragmatic trial framework and reviewed trial literature. Second, we aligned trial implementation and components with major health reform elements. Third, a series of stakeholder interviews and focus groups were held to understand the values, understandings, perceptions, and intentions in regard to the health reform efforts and the potential for the trial design to be nested within these efforts. Finally, a pilot was completed to test various elements prior to the study start.

Changes to the trial design from this initial set of tasks are outlined in Table 1. The PRECIS framework recommends health professional and patient autonomy over trial protocol inflexibility and prescription, and the trial was broadened to allow flexibility in quality improvement (QI) decision-making, meeting practices where they are in terms of capacity for change. The alignment with payers and other stakeholders demonstrated that certain principles could be agreed upon, but flexibility was needed and simplification was required for specific improvement. Patients focused on access and information gaps; subsequently, we included access-related high-value elements, and all trial elements were translated to patient-specific language.

**Table 1:** **Preparation steps for trial design**

**Aspect**	**Results**	**Changes made**
PRECIS (pragmatic trial) review	Initial high-value elements were too focused and prescriptive	Broadened to allow flexibility and health professional judgment
Alignment with health reform via multistakeholder panel (*N* = 12)	Incentives too complex; lack of alignment with other health reform efforts; team and clinic culture will drive results	Generated alignment document; revised intervention metrics and approach to better align with initiatives; extensive team and clinic assessment
Chronic illness patient focus group (*N* = 9)	Access to the clinic and the specific services need to be improved; information gaps about what is newly available (like care coordination) was common	Look carefully for patient experience measures that examine access and information gathering; expect different experiences from patients than reported by clinics; improve education
Insurer focus group (*N* = 22)	Each insurer had their own initiative or had a separate take on the current initiative set	Aligned principles were agreed to by payers; encourage payer alignment
Pilot clinic interviews (*N* = 6)	Expectations of the clinic too vague and time-consuming	Generated a memorandum of understanding that clinics revised and implemented

### Pilot study

A 6-month pilot at a single practice site was performed to test the various interventions and refine efforts. At the pilot site, preparation and protocol elements were tested in a series of six monthly practice facilitation visits. At each visit, an aspect of the trial design was tested for feasibility, clarity, and usability. The pilot team was interviewed for the results, and results demonstrated that the expectations were too vague and time-consuming. A memorandum of agreement was drawn up with specific expectations for both the research team and the clinic team. These included a role-based specification by clinic of time expected, use of data, and communication required to participate, as well as clear roles for researchers. The protocol visits by practice facilitators were honed to focus on key drivers of quality improvement and transformation: leadership, team-based approach, QI infrastructure, setting new improvement targets, and follow-up on previous improvement targets. Reports were assessed for comprehension and revised, and use of the IT system for study participation was reduced, allowing clinics to use their own protocols and tools when they matched the function of the IT system.

### Participants

Participants are eight primary care clinics (clusters) that are deeply engaged in health reform, are willing to use a population management registry tool, are willing to receive practice facilitation and improve their performance, and use an electronic health record (EHR) system at baseline. A goal was set to recruit a diverse set of participant clinics by ownership, location (rural versus urban), and size (from 3 to 20 providers). Patients, providers, and staff of these clinics are consented separately when their specific opinions or viewpoints are elicited (via surveys or interviews), with no penalty for non-participation. Data is collected at the clinics during facilitation sessions, from the EHR through automated extraction and from patients, providers, and staff via paper and online surveys.

### Interventions

**Figure 1:**
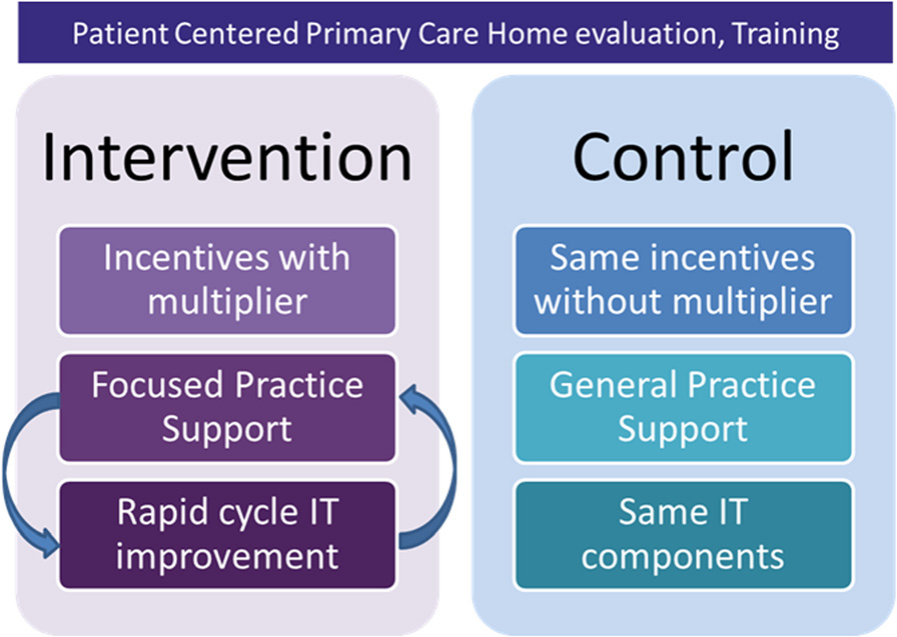
**TOPMED final trial design (see accompanying file).**

The final trial design, shown in Figure 1, is a two-arm, cluster randomized controlled trial with clustering at the clinic level and stratification by size, location, and population. We considered a factorial design and a third arm with extant patient-centered medical homes but no specific interventions (usual care), but contamination prevented a factorial design and the third arm required access to unavailable clinic data.

These components consist of three aspects aimed at the clinic level: 1) incentives with audit and feedback based on overall and incremental goal performance; 2) an HIT system called the Integrated Care Coordination Information System (ICCIS) that measured care on individual patients as well as progress towards clinic goals; and 3) tailored practice facilitation. The sole difference between the intervention and control arm is that the control arm is given free rein to address any health reform goal and the intervention arm is encouraged—both through practice facilitation and incentives—to focus their transformation efforts on achieving as many high-value elements as possible. A summary is shown in Table 2 below. Across five topic areas for high-value elements, 12 subtopics were developed into 26 specific measures that allow flexibility and increase achievement. Components such as identification of at-risk population and care management are measured by provision of care plans, offering advanced directives, and outreach to high-risk populations. Patient engagement and proactive goal setting involve self-management support and reminders. Integrating information focuses on clinical information exchange and follow-up post-utilization, while population management focuses on the use of population management tools to proactively manage metrics and certain outreach components. Finally, access was added based on patient-identified need; after-hours access and availability of providers are the identified elements from the focus group. Alignment with health reform metrics is moderate to strong. Moderate alignment was defined as some overlap with the general area of health reform, but the metric did not exist in the reform model. For instance, risk stratified care management concepts were not measures in the reviewed initiatives but rather general goals. Strong alignment means that the precise measure was or is in place elsewhere, but the benchmark was more advanced or specific. For instance, follow-up on utilization is a specific CPC metric, but the study benchmark of 70% is more aggressive.

**Table 2:** **Intervention ‘high-value’ elements**

**Element**	**Description**	**Specific example metric** ^*^	**Alignment with reform**
Identification of at-risk populations and care management	Care plan utilization	Tier 3—PCMH provides care plans to >50% of high-risk patients.	Moderate
	Advance directive utilization	Tier 3—The PCMH offers advance directives to at least 50% of patients over 65.	
Based on need	Care management outreach	Tier 2—PCMH’s care coordination outreach reaches 50% of high-risk patients.	
Patient engagement and proactive goal setting	Education and self-management resources	Tier 2—More than 10% of all unique patients are provided patient-specific education resources and self-management services.	Strong
	Reminders	Tier 3—PCMH sends appropriate reminders to at least 20% of all eligible patients.	
Integrated information and procedures across settings	Clinical information exchange	Tier 1—PCMH exchanges structured clinical information and tracks critical elements (e.g., hospitalizations).	Strong
	Utilization monitoring and follow-up	Tier 3—PCMH follows up on patient hospitalizations and ED visits 70% of the time (when they have the information).	
Population management tools	Performance data utilization	Tier 1—The PCMH uses performance data to identify opportunities for improvement and acts to improve clinical quality, efficiency, and patient experience.	Strong
	Receive and respond to electronic requests	Tier 3—The PCMH provides a response to online or electronic queries within two business days.	
Improved access	After-hours access	Tier 1—PCMH offers access to in-person care at least 12 h weekly outside traditional business hours.	Strong
	Tracking 3rd next available appointments		

*Nine metrics are solely applicable to primary care, and eight include primary care and other aspects of the health care system.

### Trial implementation and monitoring

Clinics are eligible for the same amount of incentives. Practice facilitation is offered monthly to both intervention and control since recent summary literature suggests that practice facilitation both makes it possible to transform and directly affects outcomes of trials. At each visit, clinics are free to select from a wide variety of choices based on their current needs; while the control arm has 35, the intervention arm has 12 options. Previous goals are assessed by the practice facilitator and new ones set. The IT system is available at all times, with functions of risk stratification (built in scores), care management tracking, and self-assessment of all PCMH elements available for clinical team review. The incentive reports are built into the IT system, with the intervention and control receiving a general score based on setting and meeting QI goals, and intervention receiving a multiplier based on the number of high-value elements met. The multiplier, whose maximum was 2, set 30% of the potential incentive aside for HVE achievement. Once the clinics meet a HVE threshold of 2/3 of the elements achieved, they earn up to a 10% bonus for achieving additional elements. Additional file 1 shows the proposed IT reports for intervention and control.

The process to generate the scores was tested during the pilot study. In general, effort at QI is rewarded on a 0%–100% scale; factors included in the score generation are organization around QI, leadership involvement, team function, and setting and follow-up on goals. From the practice facilitator’s notes each month, a team of three investigators review each element and decide on a total score. This score is fed back at each visit, with a summary report of overall performance versus other clinics each quarter. The pilot clinic demonstrated a strong response to the reward of effort and the competition engendered by a ‘grading’ approach; they did require significant explanation of the scoring rubric and requested the opportunity to offer feedback to get a score adjusted.

### Outcomes and measures

There are three sets of measures planned for the trial. First, primary and secondary outcomes are aligned with the triple aim for cost/utilization, experience of care, and population health, with specific expected decreases planned as clinically meaningful and to set sample size. Primary outcomes are reduction in total cost of care for patients, with a focus on hospitalization rate and ED visit rate of patients as indicator measures. A reduction in utilization up to 20% per year has been seen in some related studies, but a conservative estimate of 10% relative reduction in utilization is planned for this study. Measurement of this outcome will be completed through a state-based All Payer All Claims database, which allows for attribution of patients to a clinic based on visits to primary care, and contains all claims for that individual across insurance types. This outcome and its specific measurement are strongly related to the health reform goals, using the same metrics for success. For experience of care, the Clinician and Group Consumer Assessment of Healthcare Providers and Systems (CG-CAHPS) surveys at the patient level will be used, with an expectation of a 10% improvement in experience of care metrics closely related to the high-value elements. There are 11 composite scores, and 4 are determined to be closely aligned to the study aims: 1) providers support you in taking care of your own health; 2) providers pay attention to your mental or emotional health; 3) follow-up on results; and 4) access to care. A secondary outcome is a composite quality score at the clinic level based on standard chronic illness health reform measures used for the clinics. A 10% increase is expected in the *control* clinics, since previous work has shown that clinics often focus on common quality measures first. Table 3 outlines primary, secondary, and monitoring outcomes, along with their respective metrics and data sources.

**Table 3:** **Primary, secondary, and monitoring outcomes**

	**Metrics**	**Measurement source**	**Alignment with health reform**
*Primary outcome goals*			
Reduce utilization by 10%	*Total cost of care*	All Payer All Claims data source; *patient level*	Strong; primary goal
	Hospitalization rate per patient per month		
	ED visit rate per patient per month		
Improve experience of care by 10%	CG-CAHPS: 4/11 composite scores	Survey to 1,600 random stratified *patients*	Strong
*Secondary outcome goals*			
Improve quality of care by 10%	Composite of nine composite measures	EHR-based electronic clinical quality measures; *clinic level*	Strong: same measures used
Improve teamness, collaboration, and patient-centered care	Composites of various tools	CSQ, TDM, CPAT, and novel collaboration tool; *clinic level*	Indirect
*Monitoring goals*			
Clusters engage in quality improvement	Monthly ‘score’ based on QI progress and goal setting	Novel practice facilitation-based measure; *clinic level*	Moderate
HVE passed monthly	Monthly count of HVEs passed	HVEs derived from PCMH; *clinic level*	Strong

Other secondary outcomes include measurements at the cluster and patient level based on stakeholder suggestion. For clinics, culture, microsystem team performance, coordination and collaboration, and adaptive reserve were felt by stakeholders and seen in the literature to be crucial to measure before and after the trial; thus, we will use the clinician staff questionnaire (CSQ), the Team Development Measure (TDM), the Collaborative Practice Assessment Tool (CPAT), and a novel collaboration tool for external collaboration between clinical teams. Qualitative interviews with patients and with clinics will be performed to understand the impact of study more broadly. Monitoring goals were discussed above but required monthly assessments of clinic engagement with QI goal setting and follow-up as well as the high-value elements will provide protocol monitoring metrics. Deviations from protocol will be recorded and used to calculate protocol adherence.

### Sample size

The sample size was based on eight hypothetical clinics with an average of 10,000 patients each. The intracluster correlation was estimated at .02, and the necessary sample size to detect a change of 10% in cost and utilization over the year was <6,000 patients with average costs of $730 per month (average Medicare spend for our region) and average hospitalization rates of 14% at a power of > .80. Besides the monitoring metrics, above, no interim analyses or stopping guidelines were planned due to the low risk of the study.

### Randomization

For randomization, with a small number of clusters (8), the study requires significant work to assure baseline similarities, where possible, and analytic techniques to adjust for site-specific differences. To that end, we used a sequence allocation that was computer-generated and used matching by key characteristics to place the clustered clinics into match groups. Match criteria included organization type and location; total patient population, and the number of patients that were high risk according to a standardized risk scoring system based on comorbidities that predict hospitalization. Sequence generation was done by a random number seeded by time; matched pairs of clinics are randomly allocated by this seed to either intervention or control. For the patient experience of care surveys, patients are randomly chosen by a computer program, with oversampling of ‘high risk’ at 50% of the overall sample. The principal investigator (PI) wrote the randomization algorithm, the matching criteria, and had multiple team members approve them as fair, unbiased, and without error prior to implementation. Once the program was run, it was sent directly to the other team members without intervention by the PI.

### Blinding and consent

For blinding, incentives vary by arm and participants are shown their own incentives. To partially blind them, the terms ‘intervention’ and ‘control’ are not used, and all reports are only shown to individual clinics without noting differences between arms. Similarly, blinding for the study team (to avoid experimenter’s bias) is not possible, since the reports are seen across clinics by the team. To reduce bias, the practice facilitator is required to log all interactions and have these reviewed by a third party for time equality (same resource amounts provided to each), for minimal discussion of differences between clinics and arms, and for similar internal milestone achievement by each clinic. Blinding for the analyst is planned, with intervention and control arms labeled only as A and B, and clinic and patient identifiers are masked. Consent was sought from clinics, clinicians participating, and patients receiving the CG-CAHPS survey; other data collection is completed, done under a waiver of consent.

### Statistical methods and analysis

Descriptive analysis will compare baseline characteristics of clinics and individuals across outcomes and primary confounders (demographics, chronic illnesses, previous medical care, previous adherence to quality measures). A difference in difference calculation will compare changes in rates of outcomes for intervention versus control. Primary analysis will be a multi-level, two-stage analysis to account first for patient level and clinic level differences and then changes over time. Modeling will be done using generalized estimating equations; these can be adjusted for the level and the outcome variable specifics. The risk of selection bias by cluster is high, so to account for this, we pre-assess the population by risk of hospitalization using a standardized risk score prior to trial start. This group becomes the analytic cohort, and the size of each high-risk group is used to stratify clusters. We will generate the intracluster correlation, account for it by adjusting multivariate analysis, and perform propensity matching within the analytic cohort to assure similarity of baseline cohorts at the clinics. Overfitting will be tested using Copa’s test and model fit using Hosmer-Lemeshow, receiver operating curves, and Akaike information criterion for logistic-based models and Box-Cox test, GLM family test, Link and RESET test in addition to *R*^2^, and mean-squared error for cost-based models.

### Ethical approval and trial status

Ethical approval was granted by the Oregon Health and Science University (OHSU) Institutional Review Board. At the time of manuscript submission, the trial is ongoing. While primary data collection has concluded, we are currently collecting data from CG-CAHPS surveys and have not yet begun analysis on primary outcomes.

## Discussion

The TOPMED trial is a cluster randomized controlled trial to test whether focused quality improvement would be superior to general quality improvement in achieving cost and experience of care outcomes, especially for high-risk patients, across eight clinics. The trial has a pragmatic design, focusing on providing incentives, IT support, and practice facilitation that are intended to work in the real world of health reform and still allow choice, autonomy, and flexibility for the clinical teams.

Several trial design aspects proved challenging and have limitations. Sample size for number of clusters is low, while the patient-to-cluster ratio is high. A report by Mathematica on patient-centered medical home studies completed a quantitative analysis that concluded 200 clinics (cluster level) with 10 patients each would have significantly more power than 10 clinics with 200 patients each [13]. However, stakeholders reflected to our study team that the ability to understand what is happening in each cluster can be extraordinarily complex—a clinic is an organization made up of many different microsystems, changing processes, and subpopulations of patients who may self-select a particular microsystem based on their needs; failing to measure these microsystems leads to errors. In addition, significant resources are required to try to alter processes within a single clinic, making studies with large number of clusters expensive. Finally, measurement of processes, structure, and other aspects of performance (e.g., culture) at the cluster level (in addition to the patient level) is also resource consuming and yet may be the single most important aspect of cluster RCTs—understanding processes and culture seems to dictate success in many implementation science reviews. Blinding for a trial design like this is not wholly possible. However, partial blinding was used and independent review of facilitator performance and scoring is intended to reduce this bias. For analysis, multiple publications have discussed the frequent need for *post hoc* adjustments in analyses, including intracluster correlations, two-stage analysis, instrumental variables, and propensity scoring as ways to adjust for baseline differences in the individual patients. Although we use two-stage analysis, differences in clusters or panels may still persist and affect outcomes.

## Conclusion

The Transforming Outcomes for Patients through Medical home Evaluation and reDesign (TOPMED) trial was designed as a pragmatic cluster randomized controlled trial with community and stakeholder input. Final trial design is a two-arm trial randomized at the clinic level with matching to improve balance. Intervention and control arms both receive equal incentives, IT support and integration, and practice facilitation; Intervention is focused on a set of literature- and stakeholder-identified high-value elements, while control will be encouraged in general quality improvement activities. Outcomes will be the triple aim of cost, patient experience of care, and population health.

## Additional file

Additional file 1:
**Proposed IT reports for intervention and control arms.**

## Abbreviations

ACO: accountable care organizations
CG-CAHPS: Clinician and Group—Consumer Assessment of Healthcare Providers and Systems
CMP: Care Management Plus
CMS: Centers for Medicare and Medicaid Services
CPAT: Collaborative Practice Assessment Tool
CPC: Comprehensive Primary Care initiative
CSQ: clinician staff questionnaire
ED: emergency department
EHR: electronic health record
HIT: health information technology
HVE: high-value elements
ICCIS: Integrated Care Coordination Information System
IT: information technology
NDP: national demonstration project
PCMH: patient-centered medical homes
PI: principal investigator
PRECIS: pragmatic-explanatory continuum indicator summary
QI: quality improvement
RCT: randomized controlled trial
TDM: Team Development Measure
TOPMED: Transforming Outcomes for Patients through Medical home Evaluation & reDesign
