# Supplementary figures and images for: Study protocol: transforming outcomes for patients through medical home evaluation and redesign: a cluster randomized controlled trial to test high value elements for patient-centered medical homes versus quality improvement
Source: Implement Sci. 2015 Jan 22;10:13. doi: 10.1186/s13012-015-0204-6 (PMC4307890; doi:10.1186/s13012-015-0204-6)

**Additional file 1. Proposed IT Reports for Intervention and Control Arms**


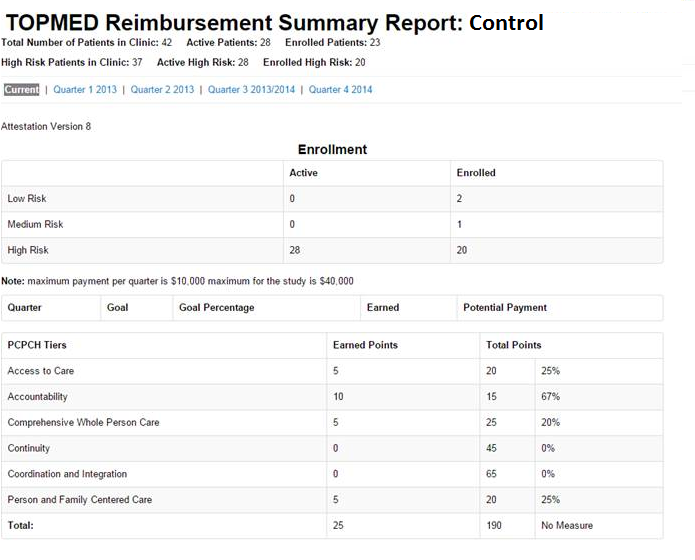


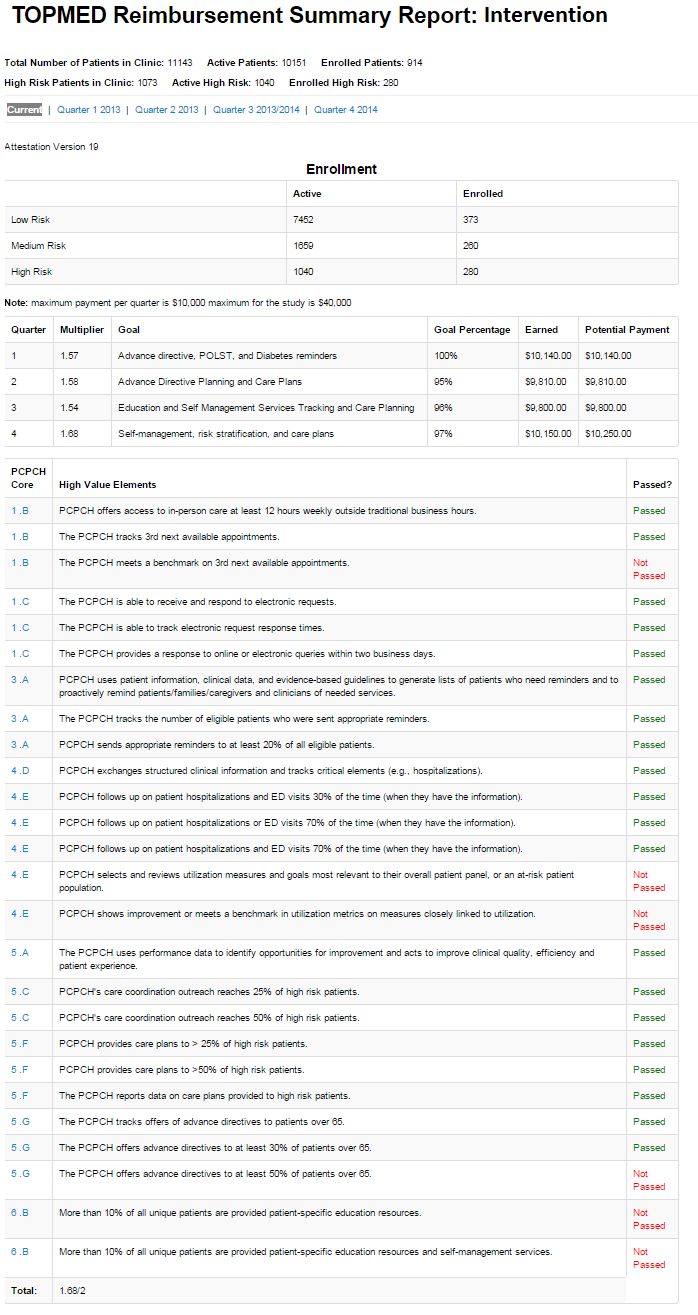

Supplement: Additional file 1: — Proposed IT reports for intervention and control arms. [file 13012_2015_204_MOESM1_ESM.docx]
